# Clinical and treatment-related risk factors for nosocomial colonisation with extensively drug-resistant *Pseudomonas aeruginosa* in a haematological patient population: a matched case control study

**DOI:** 10.1186/s12879-014-0650-9

**Published:** 2014-12-10

**Authors:** Matthias Willmann, Anna M Klimek, Wichard Vogel, Jan Liese, Matthias Marschal, Ingo B Autenrieth, Silke Peter, Michael Buhl

**Affiliations:** Institute of Medical Microbiology and Hygiene, University of Tübingen, Tübingen, Germany; German Center for Infection Research (DZIF), partner site Tübingen, Tübingen, Germany; Medical Center, Department of Hematology, Oncology, Immunology, Rheumatology & Pulmonology, University of Tübingen, Tübingen, Germany

**Keywords:** XDR, Predictors of colonisation, IMP carbapenemase, VIM carbapenemase, Clinical risk score, Clinical score construction, Matched case–control study, Conditional logistic regression

## Abstract

**Background:**

This study aimed to investigate risk factors for colonisation with extensively drug-resistant *P. aeruginosa* (XDR-PA) in immunocompromised patients and to build a clinical risk score (CRS) based on these results.

**Methods:**

We conducted a matched case–control study with 31 cases and 93 controls (1:3). Cases were colonised with XDR-PA during hospitalisation. Independent risk factors were determined using a three step conditional logistic regression procedure. A CRS was built with respect to the corresponding risk fraction of each risk factor, and its discriminatory power was estimated by receiver operating characteristic (ROC) analysis.

**Results:**

The presence of a central venous catheter (OR 7.41, P = 0.0008), the presence of a urinary catheter (OR 21.04, P < 0.0001), CRP > 10 mg/dl (OR 7.36, P = 0.0015), and ciprofloxacin administration (OR 5.53, P = 0.025) were independent risk factors. The CRS exhibited a high discriminatory power, defining a high risk population with an approximately fourteen times greater risk for XDR-PA colonisation.

**Conclusions:**

Unnecessary use of antibiotics, particularly ciprofloxacin should be avoided, and a high standard of infection control measures must be achieved when using medical devices. A CRS can be used for adaptation of the active screening culture policy to the local setting.

**Electronic supplementary material:**

The online version of this article (doi:10.1186/s12879-014-0650-9) contains supplementary material, which is available to authorized users.

## Background

The opportunistic human pathogen *Pseudomonas aeruginosa* is among the most common bacteria in health-care associated infections in Europe [1]. Severe invasive disease, particularly with multidrug-resistant strains, involves high mortality rates [2],[3]. Early detection of carriers in high-risk patients is a crucial requirement to reduce spread of resistant strains and to administer appropriate empirical treatment in case the pathogen becomes invasive. On that account there is an essential need for a comprehensive knowledge of risk factors for nosocomial colonisation with resistant *P. aeruginosa*.

Prior exposures to antimicrobials or medical devices are common risk factors reported [4]-[6] but these studies are heterogeneous in terms of design, patient population and definition of multidrug resistance. The latter issue is likely to be improved due to the publication of an international expert proposal that defined bacterial resistance profiles on the basis of epidemiological relevant antimicrobial categories [7]. The extensively drug-resistant *P. aeruginosa* (XDR-PA) that remains susceptible to a maximum of two classes of antimicrobials is clinically highly relevant due to the limited treatment options, its frequent isolation from ICU patients [8] and the recently observed international spread [9]. Risk factors have been primarily determined for invasive disease with XDR-PA [10]-[12] but predictors of patient colonisation have not yet been described.

In this study, we investigated potential risk factors for nosocomial XDR-PA colonisation in a haematological patient population. Consecutively, these results were used to construct a clinical risk score for the identification of patients at high risk for nosocomial XDR-PA colonisation, and the relative merit of this score as tool for efficient structuring of a local active screening culture policy in an endemic setting with XDR-PA was discussed.

## Methods

### Setting

The study was performed on the wards of the Department of Haematology in a 1500-bed tertiary teaching hospital in Tübingen, Germany. There are 80 beds at the department for the treatment of patients having various haematological-oncological conditions, such as leukaemia, lymphoma and multiple myeloma. Stem cell transplantations are regularly performed. One ward is an intensive care unit with single rooms. Routine screening for *P. aeruginosa* was carried out at admission and weekly thereafter. The screening involved rectal and pharyngeal swabs. Other diagnostic cultures were performed according to clinical status. The study is reported in conformity with the STrengthening the Reporting of OBservational studies in Epidemiology (STROBE) guidelines [13]. The study has been approved by the local research ethics committee of the University of Tübingen (reference number: 659/2012R).

### Study design, patients and definitions

This matched case–control study was conducted from January 2010 to December 2013. Adult patients (≥ 18 years) hospitalised > 48 h were considered eligible. Designation as case patient was based on the acquisition of a new hospital-acquired colonisation with an extensively drug-resistant *P. aeruginosa* (XDR-PA). XDR-PA were considered as hospital-acquired if they were diagnosed >48 h after admission. XDR-PA was defined according to the CDC/ECDC criteria [7]. The following antimicrobials were tested at our center: gentamicin, tobramycin, amikacin, piperacillin, piperacillin-tazobactam, ceftazidime, cefepime, ciprofloxacin, levofloxacin, meropenem, aztreonam, fosfomycin, and colistin. Intermediately susceptible isolates were considered resistant. The control group was composed of patients with either negative screening cultures for *P. aeruginosa* or of patients from whom a Non-XDR-*P. aeruginosa* was isolated. Controls were matched to cases for calendar time (quarters) and ward, and three controls were recruited for each case.

Time at risk was defined as time span between admission and new colonization with XDR-PA for cases, and as time span between admission and the last XDR-PA negative screening culture during hospitalisation for controls. According to the criteria mentioned above the minimum time at risk was three days. The primary exposure of interest was administration of antimicrobial agents. Furthermore, the length of administration (in antibiotic-days) was recorded as well as the total dose, converted into defined daily doses (DDD) conformable to the 2014 World Health Organization (WHO) anatomical therapeutic chemical (ATC) classification system [14]. Investigated antibiotics were given per os and/or intravenously and included cephalosporins (cefuroxime, ceftazidime and cefepime), quinolones (levofloxacin and ciprofloxacin), meropenem, piperacillin-tazobactam, aminoglycosides (gentamicin, tobramycin and amikacin), cotrimoxazole, macrolides (erythromycin, clarithromycin and azithromycin), doxycycline, metronidazole, vancomycin, rifampicin, clindamycin, flucloxacillin, and aztreonam.

Patient files were reviewed by medically trained staff. Clinical data included time at risk; antibiotic treatment; age; sex; length of ICU stay, infectious diseases not caused by *P. aeruginosa* (IDNPA); baseline diseases; immunosuppression, such as neutropenia (< 1000 cells/μl) and/or HIV and/or immunosuppressive chemotherapy within the previous two month (anti-inflammatory monoclonal antibody and anti-cancer drugs) and/or receipt of steroids (prednisolon ≥ 10 mg/daily or equivalent dose); Charlson comorbidity score at admission [15]; simplified acute physiology score II (SAPS II) at admission (+ 48 h) [16]; exposure to medical devices; baseline laboratory parameters during time at risk; and prior room occupation by a case patient (within 30 days and 6 weeks).

### Laboratory-based testing

Material from rectal and pharyngeal swabs was inoculated on cetrimide agar (Cetrimide Agar Base 285420, Becton, Dickinson and Company, Heidelberg, Germany). Species identification was performed by a linear MALDI-TOF mass spectrometer (AXIMA Assurance, bioMérieux, Marcy l’Etoile, France), supplemented by Vitek 2 system identification (bioMérieux, Marcy l’Etoile, France). Antimicrobial susceptibility testing was carried out with the Vitek 2 system (bioMérieux, Marcy l’Etoile, France) and interpreted following EUCAST guidelines [17].

The simultaneous detection of *bla*_VIM_ and *bla*_IMP_ genes was performed with a multiplex PCR according to a protocol described elsewhere [18]. The VIM and IMP genes were entirely sequenced using the primer pairs IMP-A–IMP-B or VIM2004A-VIM2004B in combination with the class 1 integron primer pair 5CS and 3CS or alternatively VIM-2SQR [18],[19]. Multilocus sequence typing (MLST) was conducted according to the instructions on the *P. aeruginosa* website (http://pubmlst.org/paeruginosa/).

### Statistical analysis

D’Agostino’s K-squared test was employed to check continuous variables for normality. For skewed data, medians and interquartile ranges (IQR) were provided. However, median values and IQR were 0 for several continuous variables due to their skewed distribution. In such a case, the mean and range were presented. The chi-squared test or – when appropriate – the Fisher’s exact test were performed to compare differences in proportion.

Odds ratios for XDR-PA colonisation were calculated for all variables with a minimum exposure of n = 10. Conditional logistic regression was used to calculate crude odds ratios for patient-related clinical variables. Any patient-related variable with a P-value of < 0.1 in the univariate analysis was included in the Step I multivariate conditional logistic regression model and retained when the P-value was < 0.05 using a backward stepwise elimination procedure. A Step II model was built by adding treatment-related variables (antibiotic use, length, total dose) one at a time to the final Step I model using conditional logistic regression. Length of administration and total dose of an antibiotic were investigated as quantitative variables to ensure that time- or dose-dependent effects are not missed. Treatment-related variables with a P-value < 0.1 in the Step II model were subsequently added to the patient-related variables from the Step I model, forming a final Step III multivariate conditional logistic regression model. Variables were retained when the P-value was < 0.05 using a backward stepwise elimination procedure as for the Step I model. The final Step III model contained patient- and treatment-related variables that were independent risk factors for an XDR-PA colonisation. Antibiotic use, length of use and total dose were investigated in separate Step III models to prevent biased results due to collinearity. Potential interactions were investigated using the likelihood ratio test.

For the construction of the clinical risk score (CRS) we have added up the adjusted odds ratios of all independent risk factors from the final Step III model and calculated the risk fraction according to their proportion of the total risk. Score points were allocated to each risk factor as per risk fraction value. The CRS is comprised of a total of 100 points. The discriminatory power of the CRS as well as a suitable cutoff to distinguish between patients with a high and low risk of being colonised with an XDR-PA was estimated by a receiver-operating characteristic (ROC) analysis.

A P-value < 0.05 (two-sided) was deemed statistically significant. All analyses were carried out by using Stata version 12.0 (Stat Corp., College Station, TX, USA).

## Results

A total of 124 patients (31 cases and 93 controls) were included in the risk factor analysis. Twenty-three case patients (74.2%) developed a rectal and eight case patients (25.8%) a pharyngeal XDR-PA colonisation. All XDR-PA strains were non-susceptible to piperacillin-tazobactam, ceftazidime, ciprofloxacin, meropenem, and aztreonam. Five XDR-PA (16.1%) were susceptible to aminoglycosides. A subset of 14 XDR-PA strains with identical susceptibility pattern was molecularly characterized. Twelve strains carried an IMP-8 gene and belonged to MSLT type 308 while two strains belonged to the MLST type 233 and haboured a VIM-2 gene, indicating the presence of at least two endemic strains in our setting. Sixteen control patients (17.2%) were colonised with a Non-XDR-PA during their hospital stay. The remaining 77 control patients (82.8%) were not colonized with *P. aeruginosa*. In-hospital mortality was 12.9% (4/31) for cases compared to 2.2% (2/93) for the controls (P = 0.016).

Baseline characteristics and crude odds ratios are presented in Table 1. While a number of patient-related variables seemed to increase the risk for XDR-PA colonisation, only three variables turned out to be independent risk factors in the Step I multivariate model: presence of a central venous catheter, presence of an urinary catheter, and CRP during time at risk > 10 mg/dl (Additional file 1: Table S1). Subsequently, the effect of treatment-related variables was investigated. Generally, case patients were more likely to have received antibiotics compared to controls (100% vs. 78.5%, P = 0.003). To gain more specific results, all treatment-related variables were added to the Step I model one at a time (Step II models). The administration of ciprofloxacin and ceftazidime as well as the administration of more than three different antibiotics during the time at risk were associated with the risk of XDR-PA colonisation in these Step II models (Table 1). However, only the consumption of ciprofloxacin appeared to be an independent risk factor in the final Step III model (Table 2). Of note, an increase of risk with a higher total dose or a longer administration of ciprofloxacin was not observed. The full list with results of treatment-related variables from all Step II models and the findings from all Step III models are shown in the Additional file 1: Table S2 and S3, respectively.

**Table 1:** **Baseline characteristics, comorbidities, laboratory and Step II treatment parameters of 124 patients: odds ratios for risk of XDR-PA colonisation**

Parameter	Cases (n = 31)	Controls (n = 93)	Crude OR (95% CI)	P-value
*Basic parameters*				
Age, median (IQR), years	56 (48–68)	60 (51–70)	0.9835 (0.9539 - 1.0141)^‡^	0.28
Female sex, %	11 (35.5%)	20 (21.5%)	1.19 (0.53 - 2.65)	0.68
Admission from home, %	26 (83.9%)	83 (89.3%)	0.61 (0.18 - 2.01)	0.42
Stay on ICU, %	12 (38.7%)	25 (26.9%)	6.23 (1.16 - 33.4)	0.024
Length of ICU stay, mean (range), days	10.58 (0–71)	9.66 (0–80)	1.0055 (0.9747 - 1.0374)^‡^	0.73
Time at risk > 14 days, %	19 (61.3%)	39 (41.9%)	2.54 (1.01 - 6.34)	0.04
IDNPA, %	17 (54.8%)	20 (21.5%)	5.47 (1.95 - 15.37)	0.0004
*Comorbid conditions*				
Immune suppression, %	30 (96.8%)	79 (85%)	5.35 (0.67 - 42.5)	0.05
Charlson Comorbidity Score, median (IQR)	2 (2–4)	2 (2–4)	1.13 (0.91 - 1.41)	0.28
Diabetes, %	5 (16.1%)	16 (17.2%)	0.92 (0.3 - 2.8)	0.89
Cardiovascular disease, %	15 (48.4%)	37 (39.8%)	1.44 (0.62 - 3.3)	0.4
*Patient’s clinical record*				
SAPS II, mean (range)	27.97 (13–44)	26.32 (6–51)	1.0234 (0.9752 - 1.074)^‡^	0.35
Neutropenia (<1000 cells/μl), %	22 (71%)	51 (54.8%)	2.13 (0.86 - 5.31)	0.095
Length of Neutropenia, median (IQR), days	9 (0–18)	2 (0–16)	1.0239 (0.9897 - 1.0592)^‡^	0.17
Non-invasive ventilation, %	9 (29%)	24 (25.8%)	1.2 (0.46 - 3.13)	0.71
CVC, %	21 (67.7%)	40 (43%)	3.48 (1.35 - 9)	0.008
Length of CVC, mean (range), days	14.13 (0–59)	11.13 (0–86)	1.0155 (0.9867 - 1.0452)^‡^	0.3
Urinary catheter, %	13 (41.9%)	8 (8.6%)	6.75 (2.38 - 19.17)	0.0001
Length of urinary catheter, mean (range), days	4.03 (0–20)	0.98 (0–23)	1.1159 (1.0266 - 1.2129)^‡^	0.005
Room preoccupied by case (30 days), %	4 (12.9%)	7 (7.5%)	1.46 (0.48 - 8.4)	0.35
Room preoccupied by case (6 weeks), %	4 (12.9%)	9 (9.7%)	1.41 (0.38 - 5.22)	0.61
*Most pathological laboratory parameter during time at risk*				
White blood cell count, median (IQR), cells/μl	1260 (330–3170)	2060 (360–5720)	0.99998 (0.99993 - 1.00004)^‡^	0.73
Neutrophils, median (IQR), cells/μl	320 (20–1076)	1090 (50–3383)	0.9999 (0.9998 - 1)^‡^	0.14
Platelet count < 50,000 cells/μl, %	21 (67.7%)	44 (47.3%)	2.55 (1.04 - 6.28)	0.04
Creatinine, median (IQR), mg/dl	1.1 (0.8 - 1.4)	1 (0.8 - 1.2)	1.27 (0.85 - 1.88)^‡^	0.24
CRP > 10 mg/dl, %	21 (67.7%)	32 (34.4%)	5.29 (1.88 - 14.89)	0.0005
Step II treatment variables*	*Cases (n = 31)*	*Controls (n = 93)*	*OR (95% CI)*	*P-value*
Ceftazidime use, %	10 (32.3%)	5 (5.4%)	4.28 (0.74 - 24.77)	0.09
Ciprofloxacin use, %	8 (25.8%)	11 (11.8%)	5.53 (1.11 - 27.53)	0.025
NDA > 3, %	20 (64.5%)	27 (29%)	4.35 (0.87 - 21.68)	0.06

^‡^Per 1 unit increase.

*All antibiotics with a P < 0.1 in the Step II models are listed.

XDR-PA, extensively drug-resistant *Pseudomonas aeruginosa*; IQR, interquartile range; ICU, intensive care unit; IDNPA, infectious diseases not caused by *Pseudomonas aeruginosa*; SAPS II, Simplified Acute Physiology Score II; CVC, central venous catheter; CRP, C-reactive protein; DDD, defined daily dose; NDA, number of different antibiotics during time at risk; 95% CI, 95% confidence interval.

**Table 2:** **Multivariate analysis (Step III): Odds ratios for risk of XDR-PA colonisation**

Variable	OR (95% CI)	P-value
Clinical parameters*		
Central venous catheter	7.41 (1.98 - 27.68)	0.0008
Urinary catheter	21.04 (3.67 - 120.57)	<0.0001
CRP > 10 mg/dl	7.36 (1.81 - 29.85)	0.0015
*Treatment parameters – drug*		
Ciprofloxacin use	5.53 (1.11 - 27.53)	0.025
Ceftazidime use	1.9 (0.22 - 16.43)	0.56
NDA > 3	2.22 (0.36 - 13.65)	0.39
*Treatment parameters – length of treatment*		
Ciprofloxacin, antibiotic-days	1.01 (0.88 - 1.16)^‡^	0.88
*Treatment parameters – total dosage*		
Ciprofloxacin total dose, DDD	1.01 (0.87 - 1.17)^‡^	0.91

*The results shown are based on the final model with the treatment-related variables for use of antibiotics.

^‡^Per 1 unit increase.

XDR-PA, extensively drug-resistant *Pseudomonas aeruginosa*; CRP, C-reactive protein; NDA, number of different antibiotics during time at risk; DDD, defined daily dose; 95% CI, 95% confidence interval.

The four independent risk factors were used to build a clinical risk score. The CRS consists of 100 points, and points were allocated according to the risk fraction of each factor (Table 3). The presence of a urinary catheter was given slightly lower points than indicated by rounding the risk fraction due to the large 95% confidence interval in the final Step III model. The discriminatory power of the CRS was assessed by a ROC analysis. The area under the ROC curve (AUC) was 0.83 (95% confidence interval: 0.75 – 0.91). A cutoff was chosen to differentiate the patients in those with a higher risk of XDR-PA colonisation (high risk group) and those with a lower risk (low risk group). Patients with a CRS ≥ 36 points have an odds of XDR-PA colonisation of 1.29, thus associated with the high risk group. On the other hand, patients with a CRS < 36 points have an odds of XDR-PA colonisation of only 0.12 and thus belong with the low risk group. The risk of being a case is approximately fourteen times greater in the high risk group (conditional logistic regression, OR 14; 95% confidence interval: 4.1 – 47.9; P < 0.0001).

**Table 3:** **Buildup and structure of the clinical risk score**

Score variable	OR (step III)	Risk fraction (%)	Points
Central venous catheter	7.41	17.9%	18
Urinary catheter	21.04	50.9%	50
CRP > 10 md/dl	7.36	17.8%	18
Ciprofloxacin use	5.53	13.4%	14

XDR-PA, extensively drug-resistant *Pseudomonas aeruginosa*; CRP, C-reactive protein.

## Discussion

Our study aimed to investigate potential risk factors of XDR-PA colonisation in a haematological patient population and to set up a clinical risk score to differentiate between patients with a higher and lower risk of XDR-PA acquisition.

The primary exposure of interest was the administration of antimicrobial agents as this is one of the major concerns for the acquisition of resistant strains. We found the use of ciprofloxacin to be an independent risk factor. Ciprofloxacin is used in our institution for prophylaxis in neutropenic patients. The total dose and time span of administration did not play a role. These results are in line with previous reports for multidrug-resistant *P. aeruginosa* (MDR-PA) acquisition in different settings [5],[20],[21]. In contrast, ceftazidime administration was not independently associated with XDR-PA colonisation despite a tendency of being a risk factor in the corresponding Step II model (OR 4.28, P = 0.09). The hospital-wide use of cephalosporins was previously correlated with the incidence of XDR-PA in a multivariate time series analysis and is thus likely to exist for the hospital setting rather than for the Department of Haematology alone [22]. However, an independent association between ceftazidime administration and XDR-PA colonisation in our haematological patient population cannot be excluded due to limitations in the study power of the present investigation. Moreover, the inclusion of patients with Non-XDR-PA colonisation into the control group might have led to a selection bias according to Harris et al. [23],[24]. In the present study, this should have caused only a minor bias if at all due to the low proportion of these patients among the control group (17.2%). Regarding these patients as a part of the source population from which case patients could arise prevents a general selection bias and retains the internal and external validity of our study [25].

Another interesting discovery was an increase in risk when more than three different antibiotics were administered during hospitalisation (OR 4.35, P = 0.06). Although this result was not confirmed in the final Step III model and must thus be interpreted with caution, it suggests that the use of different antibiotics could ease an at least temporary establishment of XDR-PA, possibly due to a broader and more destructive impact on the gut or respiratory tract flora compared to treatment with fewer agents.

Surprisingly, a relatively low risk fraction for ciprofloxacin use (13.4%) was observed when compared with independent patient-related risk factors. Primarily the use of medical devices had a major impact on the risk of XDR-PA colonisation (central venous catheter and urinary catheter; joint risk fraction 68.8%, Table 3). A recent meta-analysis has shown that medical devices are leading risk factors for the acquisition of carbapenem-resistant *P. aeruginosa* [6]. This indicates that invasive procedures like insertion of catheters by medical personnel can contribute to the spread and sustainment of resistance in the hospital. Interestingly, in contrast to a previous report we have not seen that prior occupation of a room with a case patient has increased the risk for subsequent patients [26]. This supports the hypothesis that medical personnel represent – in terms of XDR-PA acquisition - a greater risk for the patient than environmental contamination. However, the results of the univariate analysis suggest that with every urinary catheter day evolves a stepwise increase in the odds ratio for XDR-PA colonisation of approximately 11% (P = 0.005), indicating that having a urinary catheter could lead to a patient behaviour, special care or exposure to unknown environmental factors that further increase the acquisition risk.

A C-reactive protein > 10 mg/dl was identified as another independent and patient-related risk factor. It is not within the scope of our study to give a comprehensive explanation for this finding, but it can be speculated whether this reflects a pro-inflammatory situation or a concomitant infectious disease that impairs the protective mucosal barrier and could thus promote a more effective adherence of the pathogen.

We have found a significantly higher in-hospital mortality among case patients compared to controls (12.9% versus 2.2%, P = 0.016). But this results need to be interpreted with caution since we have not directly investigated the impact of XDR-PA colonisation on mortality. However, it strengthens the findings of a previous study in our setting where mortality was significantly different between patients with bloodstream infection due to metallo-β-lactamase (MBL) producing *P. aeruginosa* or Non-MBL producing *P. aeruginosa* ( 61% versus 34%) [27]. Additionally, Tacconelli et al. have reported that 9% of patients with a new hospital-acquired colonisation with an antibiotic resistant bacterium developed subsequently an infection due to the same pathogen [4]. Thus, identifying the patients’ carrier status could improve chances to choose an appropriate empirical treatment when necessary, particularly in an institution with a relevant incidence of multidrug-resistant pathogens.

**Figure 1:**
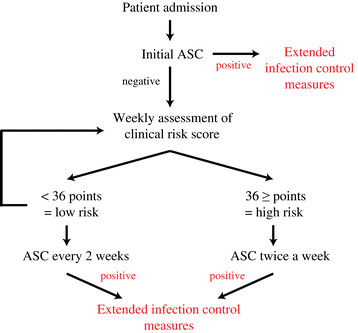
**Flow chart for a locally adapted active screening culture (ASC) strategy.** Patients once classified as high risk patients for XDR-PA colonisation remain their status until discharge und are screened twice a week.

The weekly rectal and pharyngeal screening for *P. aeruginosa* was introduced in our Department of Haematology in response to an increase in the incidence of MDR- and XDR-PA. It is a reasonable assumption that active screening cultures (ASC) facilitate an early detection of carrier status that should be followed by appropriate infection control measures when an MDR- or XDR-PA is found. On the other hand, there is no evidence at the moment that ASC is effective in decreasing the incidence of targeted organisms in the long-term, and thus it is only recommended as an additional procedure [28]. However, ASC might be more effective if the screening strategy is adapted to the local setting. The differentiation into low and high risk groups according to local risk factors and a subsequent modification of the screening frequency for both groups could be an approach to efficiently allocate resources and focus on the patients who could benefit the most from ASC. Our CRS exhibits a high discriminatory power (AUC = 0.83) with an approximately fourteen times greater risk for nosocomial colonisation with XDR-PA in the high risk group. An example of how a weekly screening strategy for all patients can be adjusted to the local setting by using such a CRS is shown in Figure 1.

We are aware that our study has several limitations. The CRS is derived from the odds ratios of significant risk factors in our study population. Its true performance would need to be evaluated on another patient cohort in the same setting, preferably with a prospective design. In addition, the study power could have been too low to reveal moderately associated risk factors. Also, this is a single center study and results are usually not transferable to other settings. However, it must be stated that even results of multicenter studies as well as recommendations from generally accepted guidelines can turn out as inappropriate. An example are the results from a research group in eastern China who found the American Thoracic Society (AST) guidelines criteria not reliable for the prediction of multidrug-resistant organisms in their hospital [29]. For this reason, we recommend for any setting i) to identify the locally most common resistant pathogens, ii) to investigate the most relevant risk factors for acquisition, and iii) to adapt the local infection control policy according to the observed risk factors, for instance in the context of a local CRS as suggested by our work.

## Conclusion

We found ciprofloxacin use as well as patient-related factors to independently increase the risk for XDR-PA colonisation. The results indicate that unnecessary administration of antibiotics, specifically ciprofloxacin should be avoided and that a high infection control standard must be accomplished when medical devices are used. Additionally, a switch to alternative regimes for prophylaxis in neutropenic patients is a recommendable option. Identified risk factors can be applied to adapt local screening strategies for an early detection of resistant organisms with a subsequently more efficient prevention of their spread and administration of appropriate empirical treatment if necessary.

## Additional file

## Electronic supplementary material

Additional file 1: Additional tables including details from the uni- and multivariate analysis which are not shown in the manuscript text. (PDF 296 KB)

Below are the links to the authors’ original submitted files for images.

Authors’ original file for figure 1

## References

[1] European Centre for Disease Prevention and Control: Point prevalence survey of health care associated infections and antimicrobial use in European acute care hospitals. 2013 [], [http://www.ecdc.europa.eu/en/publications/Publications/healthcare-associated-infections-antimicrobial-use-PPS.pdf]

[2] Aloush V, Navon-Venezia S, Seigman-Igra Y, Cabili S, Carmeli Y (2006). Multidrug-resistant Pseudomonas aeruginosa: risk factors and clinical impact. Antimicrob Agents Chemother.

[3] Tumbarello M, Repetto E, Trecarichi EM, Bernardini C, De Pascale G, Parisini A, Rossi M, Molinari MP, Spanu T, Viscoli C, Cauda R, Bassetti M (2011). Multidrug-resistant Pseudomonas aeruginosa bloodstream infections: risk factors and mortality. Epidemiol Infect.

[4] Tacconelli E, De Angelis G, Cataldo MA, Mantengoli E, Spanu T, Pan A, Corti G, Radice A, Stolzuoli L, Antinori S, Paradisi F, Carosi G, Bernabei R, Antonelli M, Fadda G, Rossolini GM, Cauda R (2009). Antibiotic usage and risk of colonization and infection with antibiotic-resistant bacteria: a hospital population-based study. Antimicrob Agents Chemother.

[5] Nouer SA, Nucci M, de-Oliveira MP, Pellegrino FL, Moreira BM (2005). Risk factors for acquisition of multidrug-resistant Pseudomonas aeruginosa producing SPM metallo-beta-lactamase. Antimicrob Agents Chemother.

[6] Holt AF VI't, Severin JA, Lesaffre EM, Vos MC (2014). A systematic review and meta-analyses show that carbapenem use and medical devices are the leading risk factors for carbapenem-resistant Pseudomonas aeruginosa. Antimicrob Agents Chemother.

[7] Magiorakos AP, Srinivasan A, Carey RB, Carmeli Y, Falagas ME, Giske CG, Harbarth S, Hindler JF, Kahlmeter G, Olsson-Liljequist B, Paterson DL, Rice LB, Stelling J, Struelens MJ, Vatopoulos A, Weber JT, Monnet DL (2012). Multidrug-resistant, extensively drug-resistant and pandrug-resistant bacteria: an international expert proposal for interim standard definitions for acquired resistance. Clin Microbiol Infect.

[8] Gomez-Zorrilla S, Camoez M, Tubau F, Periche E, Canizares R, Dominguez MA, Ariza J, Pena C (2014). Antibiotic pressure is a major risk factor for rectal colonization by multidrug-resistant pseudomonas aeruginosa in critically ill patients. Antimicrob Agents Chemother.

[9] Edelstein MV, Skleenova EN, Shevchenko OV, D'Souza JW, Tapalski DV, Azizov IS, Sukhorukova MV, Pavlukov RA, Kozlov RS, Toleman MA, Walsh TR (2013). Spread of extensively resistant VIM-2-positive ST235 Pseudomonas aeruginosa in Belarus, Kazakhstan, and Russia: a longitudinal epidemiological and clinical study. Lancet Infect Dis.

[10] Samonis G, Vardakas KZ, Kofteridis DP, Dimopoulou D, Andrianaki AM, Chatzinikolaou I, Katsanevaki E, Maraki S, Falagas ME (2014). Characteristics, risk factors and outcomes of adult cancer patients with extensively drug-resistant Pseudomonas aeruginosa infections. Infection.

[11] Pena C, Gomez-Zorrilla S, Suarez C, Dominguez MA, Tubau F, Arch O, Oliver A, Pujol M, Ariza J (2012). Extensively drug-resistant Pseudomonas aeruginosa: risk of bloodstream infection in hospitalized patients. Eur J Clin Microbiol Infect Dis.

[12] Liew YX, Tan TT, Lee W, Ng JL, Chia DQ, Wong GC, Kwa AL (2013). Risk factors for extreme-drug resistant Pseudomonas aeruginosa infections in patients with hematologic malignancies. Am J Infect Control.

[13] Vandenbroucke JP, von Elm E, Altman DG, Gotzsche PC, Mulrow CD, Pocock SJ, Poole C, Schlesselman JJ, Egger M (2007). Strengthening the reporting of observational studies in epidemiology (STROBE): explanation and elaboration. PLoS Med.

[14] WHO: Guidelines for ATC classification and DDD assignment, 2014. WHO Collaborating Centre for Drug Statistics Methodology; 2013 [], [http://www.whocc.no/filearchive/publications/2014_guidelines.pdf]

[15] Charlson ME, Pompei P, Ales KL, MacKenzie CR (1987). A new method of classifying prognostic comorbidity in longitudinal studies: development and validation. J Chronic Dis.

[16] Le Gall JR, Lemeshow S, Saulnier F (1993). A new simplified acute physiology score (SAPS II) based on a european/north american multicenter study. JAMA.

[17] Breakpoint tables for interpretation of MICs and zone diameters. [], [http://www.eucast.org/fileadmin/src/media/PDFs/EUCAST_files/Breakpoint_tables/Breakpoint_table_v_2.0_120221.pdf]

[18] Pitout JD, Gregson DB, Poirel L, McClure JA, Le P, Church DL (2005). Detection of Pseudomonas aeruginosa producing metallo-beta-lactamases in a large centralized laboratory. J Clin Microbiol.

[19] Lee MF, Peng CF, Hsu HJ, Chen YH (2008). Molecular characterisation of the metallo-beta-lactamase genes in imipenem-resistant Gram-negative bacteria from a university hospital in southern Taiwan. Int J Antimicrob Agents.

[20] Montero M, Sala M, Riu M, Belvis F, Salvado M, Grau S, Horcajada JP, Alvarez-Lerma F, Terradas R, Orozco-Levi M, Castells X, Knobel H (2010). Risk factors for multidrug-resistant Pseudomonas aeruginosa acquisition: impact of antibiotic use in a double case–control study. Eur J Clin Microbiol Infect Dis.

[21] Paramythiotou E, Lucet JC, Timsit JF, Vanjak D, Paugam-Burtz C, Trouillet JL, Belloc S, Kassis N, Karabinis A, Andremont A (2004). Acquisition of multidrug-resistant Pseudomonas aeruginosa in patients in intensive care units: role of antibiotics with antipseudomonal activity. Clin Infect Dis.

[22] Willmann M, Marschal M, Holzl F, Schroppel K, Autenrieth IB, Peter S (2013). Time series analysis as a tool to predict the impact of antimicrobial restriction in antibiotic stewardship programs using the example of multidrug-resistant Pseudomonas aeruginosa. Antimicrob Agents Chemother.

[23] Harris AD, Karchmer TB, Carmeli Y, Samore MH (2001). Methodological principles of case–control studies that analyzed risk factors for antibiotic resistance: a systematic review. Clin Infect Dis.

[24] Harris AD, Samore MH, Lipsitch M, Kaye KS, Perencevich E, Carmeli Y (2002). Control-group selection importance in studies of antimicrobial resistance: examples applied to Pseudomonas aeruginosa, Enterococci, and Escherichia coli. Clin Infect Dis.

[25] Elwood M (2007). Critical Appraisal of Epidemiological Studies and Clinical Trials.

[26] Nseir S, Blazejewski C, Lubret R, Wallet F, Courcol R, Durocher A (2011). Risk of acquiring multidrug-resistant Gram-negative bacilli from prior room occupants in the intensive care unit. Clin Microbiol Infect.

[27] Willmann M, Kuebart I, Marschal M, Schroppel K, Vogel W, Flesch I, Markert U, Autenrieth IB, Holzl F, Peter S (2013). Effect of metallo-beta-lactamase production and multidrug resistance on clinical outcomes in patients with Pseudomonas aeruginosa bloodstream infection: a retrospective cohort study. BMC Infect Dis.

[28] Tacconelli E, Cataldo MA, Dancer SJ, De Angelis G, Falcone M, Frank U, Kahlmeter G, Pan A, Petrosillo N, Rodriguez-Bano J, Singh N, Venditti M, Yokoe DS, Cookson B (2014). ESCMID guidelines for the management of the infection control measures to reduce transmission of multidrug-resistant Gram-negative bacteria in hospitalized patients. Clin Microbiol Infect.

[29] Xie J, Ma X, Huang Y, Mo M, Guo F, Yang Y, Qiu H (2014). Value of American Thoracic Society guidelines in predicting infection or colonization with multidrug-resistant organisms in critically ill patients. PLoS One.

