# Supplementary material for: Clinical and treatment-related risk factors for nosocomial colonisation with extensively drug-resistant Pseudomonas aeruginosa in a haematological patient population: a matched case control study
Source: BMC Infect Dis. 2014 Dec 10;14:650. doi: 10.1186/s12879-014-0650-9 (PMC4266216; doi:10.1186/s12879-014-0650-9)
Supplement: Supplementary file 1 — Additional file 1: Additional tables including details from the uni- and multivariate analysis which are not shown in the manuscript text. (PDF 296 KB) [file 12879_2014_650_MOESM1_ESM.pdf]

Supplementary Materials for

Clinical and treatment-related risk factors for nosocomial colonisation with extensively drug-resistant *Pseudomonas aeruginosa* in a haematological patient population

Authors: Matthias Willmann,[1,2*] Anna M Klimek,[1,2] Wichard Vogel,[3] Jan Liese,[1,2] Matthias Marschal,[1,2] Ingo B Autenrieth,[1,2] Silke Peter,[1,2] and Michael Buhl.[1,2]

[1]Institute of Medical Microbiology and Hygiene, University of Tübingen, Tübingen, Germany.

[2]German Center for Infection Research (DZIF), partner site Tübingen, Tübingen, Germany.

[3]Medical Center, Department of Hematology, Oncology, Immunology, Rheumatology & Pulmonology, University of Tübingen, Tübingen, Germany.

[*]Corresponding author

Contact details of corresponding author:
Dr. Matthias Willmann, MD, MSc, DTM&H
Institute of Medical Microbiology and Hygiene
Elfriede-Aulhorn-Str. 6
72076, Tübingen
Germany
matthias.willmann@med.uni-tuebingen.de
will80@gmx.de
Phone: +49 7071/29-81527
Fax: +49 7071/29-5440

Supplementary tables

Table S1. Step I multivariate model: Odds ratios for risk of XDR-PA colonisation

Variable	OR (95% CI)	P-value
Central venous catheter	6.17 (1.79 - 21.32)	0.0014
Urinary catheter	12.78 (3.04 – 53.72)	< 0.0001
CRP > 10 mg/dl	8.84 (2.33 - 33.54)	0.0002

XDR-PA, extensively drug-resistant *Pseudomonas aeruginosa*; CRP, C-reactive protein; 95% CI, 95% confidence interval.

Table S2. Step II treatment-related variables: Odds ratios for risk of XDR-PA colonisation

Parameter	Cases (n = 31)	Controls (n = 93)	OR (95% CI)	P-value
Use of any antibiotic, %	31 (100%)	73 (78.5%)	n.a.	0.003*
Any antibiotic, median (IQR), antibiotic-days	15 (8 - 27)	8 (1 - 24)	0.9811 (0.9448 - 1.0189)	0.32
Any antibiotics total dose, median (IQR), DDD	34.31 (11 - 56.83)	11.67 (0.5 - 43.86)	0.9949 (0.9809 - 1.0091)	0.48
Piperacillin-tazobactam use, %	20 (64.5%)	46 (49.5%)	0.89 (0.27 - 2.94)	0.85
Piperacillin-tazobactam, mean (range), antibiotic-days	5.26 (0 - 20)	3.78 (0 - 26)	0.99 (0.89 - 1.11)	0.89
Piperacillin-tazobactam total dose, mean (range), DDD	4.57 (0 - 15.43)	3.21 (0 - 19.29)	1.01 (0.88 - 1.16)	0.88
Meropenem use, %	21 (67.7%)	30 (32.3%)	3.1 (0.66 - 14.51)	0.13
Meropenem, mean (range), antibiotic-days	8.55 (0 - 66)	5.95 (0 - 59)	0.96 (0.9 - 1.02)	0.14
Meropenem total dose, mean (range), DDD	11.53 (0 - 97.5)	8.32 (0 - 85)	0.97 (0.93 - 1.01)	0.13
Ciprofloxacin use, %	8 (25.8%)	11 (11.8%)	5.53 (1.11 - 27.53)	0.025
Ciprofloxacin, mean (range), antibiotic-days	1.35 (0 - 8)	1.06 (0 - 21)	1.01 (0.88 - 1.16)	0.88
Ciprofloxacin total dose, mean (range), DDD	1.35 (0 - 12.8)	0.96 (0 - 18.25)	1.01 (0.87 - 1.17)	0.91
Levofloxacin use, %	15 (48.4%)	35 (37.6%)	1.25 (0.37 - 4.18)	0.71
Levofloxacin, mean (range), antibiotic-days	5.87 (0 - 43)	6.7 (0 - 67)	0.96 (0.9 - 1.02)	0.13
Levofloxacin total dose, mean (range), DDD	5.65 (0 - 43)	6.43 (0 - 67)	0.95 (0.89 - 1.02)	0.14
Ceftazidime use, %	10 (32.3%)	5 (5.4%)	4.28 (0.74 - 24.77)	0.09
Ceftazidime, mean (range), antibiotic-days	3.81 (0 - 25)	0.28 (0 - 11)	1.23 (1 - 1.51)	0.023
Ceftazidime total dose, mean (range), DDD	3.45 (0 - 35)	0.21 (0 - 6.25)	1.3 (0.98 - 1.71)	0.02
Cefepime use, %	2 (6.5%)	0 (0%)	n.a.	n.a.
Gentamicin use, %	2 (6.5%)	0 (0%)	n.a.	n.a.
Tobramycin use, %	0 (0%)	0 (0%)	n.a.	n.a.
Amakacin use, %	0 (0%)	0 (0%)	n.a.	n.a.
Cotrimoxazole use, %	21 (67.7%)	51 (54.8%)	1.16 (0.29 - 4.67)	0.84
Cotrimaxazole, mean (range), antibiotic-days	3.1 (0 - 10)	2.3 (0 -14)	0.99 (0.85 - 1.16)	0.92
Cotrimoxazole total dose, mean (range), DDD	1.82 (0 - 5)	1.57 (0 -23)	0.92 (0.73 - 1.16)	0.44
Erythromycin use, %	0 (0%)	0 (0%)	n.a.	n.a.
Clarithromycin use, %	6 (19.4%)	11 (11.8%)	1 (0.21 - 4.7)	0.99
Clarithromycin, mean (range), antibiotic-days	1.74 (0 - 14)	0.57 (0 - 13)	1.09 (0.9 - 1.32)	0.38
Clarithromycin total dose, mean (range), DDD	1.94 (0 - 14)	0.73 (0 - 17.5)	1.06 (0.91 - 1.25)	0.44
Azithromycin use, %	0 (0%)	0 (0%)	n.a.	n.a.
Doxycyclin use, %	1 (3.2%)	0 (0%)	n.a.	n.a.
Metronidazole use, %	10 (32.3%)	13 (14%)	1.75 (0.51 - 6)	0.37
Metronidazole, mean (range), antibiotic-days	3.48 (0 - 27)	1.28 (0 - 27)	1.02 (0.93 - 1.12)	0.67

Metronidazole total dose, mean (range), DDD	2.81 (0 - 19)	1.14 (0 - 24.8)	1.02 (0.92 - 1.13)	0.74
Vancomycin use, %	7 (22.6%)	18 (19.4%)	2.03 (0.35 - 11.88)	0.42
Vancomycin, mean (range), antibiotic-days	2.22 (0 - 24)	2.66 (0 - 46)	0.96 (0.87 - 1.06)	0.4
Vancomycin total dose, mean (range), DDD	1.65 (0 - 20.5)	1.96 (0 - 34)	0.95 (0.84 - 1.07)	0.36
Rifampicin use, %	0 (0%)	0 (0%)	n.a.	n.a.
Clindamycin use, %	4 (12.9%)	6 (6.5%)	1.13 (0.23 - 5.6)	0.88
Clindamycin, mean (range), antibiotic-days	0.97 (0 - 17)	0.38 (0 - 8)	0.97 (0.74 - 1.26)	0.82
Clindamycin total dose, mean (range), DDD	1.02 (0 - 21.3)	0.3 (0 - 7.2)	0.96 (0.74 - 1.26)	0.81
Cefuroxime use, %	3 (9.7%)	4 (4.3%)	n.a.	n.a.
Flucloxacillin use, %	1 (3.2%)	0 (0%)	n.a.	n.a.
Aztreonam use, %	0 (0%)	0 (0%)	n.a.	n.a.

* P-value was calculated using Fishers exact test.

Odds ratios for continuous variables (length and dosage of treatment) are given per 1 unit increase.

XDR-PA, extensively drug-resistant *Pseudomonas aeruginosa*; IQR, interquartile range; n.a., not assessed; DDD, defined daily dose; 95% CI, 95% confidence interval.

Table S3. Multivariate analysis (Step III): Odds ratios for risk of XDR-PA colonization

Variable	OR (95% CI)	P-value
Model 1 – drug		
Central venous catheter	7.41 (1.98 - 27.68)	0.0008
Urinary catheter	21.04 (3.67 - 120.57)	< 0.0001
CRP > 10 mg/dl	7.36 (1.81 - 29.85)	0.0015
Ciprofloxacin use	5.53 (1.11 – 27.53)	0.025
Model 2 – length of treatment		
Central venous catheter	6.17 (1.78 – 21.35)	0.0015
Urinary catheter	12.89 (3.05 – 54.55)	0.0001
CRP > 10 mg/dl	8.69 (2.25 – 33.59)	0.0003
Ciprofloxacin, antibiotic-days	1.01 (0.88 – 1.16)‡	0.88
Model 3 – total dosage		
Central venous catheter	6.17 (1.78 – 21.34)	0.0014
Urinary catheter	12.8 (3.04 – 53.87)	< 0.0001
CRP > 10 mg/dl	8.73 (2.26 – 33.68)	0.0003
Ciprofloxacin, DDD	1.01 (0.87 – 1.17)‡	0.91

‡ Per 1 unit increase.

XDR-PA, extensively drug-resistant *Pseudomonas aeruginosa*; CRP, C-reactive protein; DDD, defined daily dose; 95% CI, 95% confidence interval.
