# Supplementary figures and images for: Clinical and treatment-related risk factors for nosocomial colonisation with extensively drug-resistant Pseudomonas aeruginosa in a haematological patient population: a matched case control study
Source: BMC Infect Dis. 2014 Dec 10;14:650. doi: 10.1186/s12879-014-0650-9 (PMC4266216; doi:10.1186/s12879-014-0650-9)

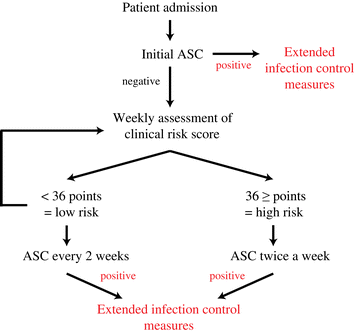

Supplement: Supplementary file 2 — Authors’ original file for figure 1 [file 12879_2014_650_MOESM2_ESM.gif]
